# Sex difference in aortic root replacement with a stentless bioprosthesis^†^

**DOI:** 10.1093/ejcts/ezaf161

**Published:** 2025-05-12

**Authors:** Hanna Dagnegård, Adriaan W Schneider, Patrick T Timmermans, Natalie Glaser, Solveig M Kolseth, Farkas Vanky, Tomas Gudbjartsson, Rune Haaverstad, Alex Cotovanu, Ulrik Sartipy, Robert J M Klautz, Morten Smerup, Jesper Hjortnaes, North Atlantic Freestyle Collaboration, Jesper Hjortnæs, Robert Klautz, Jerry Braun, Morten Smerup, Nikolaj Ihlemann, H Gustav H Thyregod, Jens T Lund, Ulrik Sartipy, Rune Haarverstad, Farkas Vanky, Tomas Gudbjartsson, Jan Brink Valentin, Søren Paaske Johnsen, Adriaan Schneider, Patrick Timmemanns, Alex Cotovanu, Hanna Dagnegård, Kirstine Bekke, Jannie Lind, Nathalie Glaser, Solveig Moss Kolseth, Jørgen Brekke Vennemo, Asbjørn Lie, Christoffer Wallén, Hörður Ingi Gunnarsson

**Affiliations:** Department of Cardiothoracic Surgery, Rigshospitalet, Copenhagen, Denmark; Department of Cardiothoracic Surgery, Leiden University Medical Center, Leiden, The Netherlands; Department of Cardiothoracic Surgery, Leiden University Medical Center, Leiden, The Netherlands; Department of Cardiology, Stockholm South General Hospital, Stockholm, Sweden; Department of Molecular Medicine and Surgery, Karolinska Institutet, Stockholm, Sweden; Department of Cardiothoracic Surgery, Haukeland University Hospital, Bergen, Norway; Department of Cardiothoracic Surgery, Linköping University Hospital, Linköping, Sweden; Department of Cardiothoracic Surgery, University Hospital, Reykjavik, Iceland; Department of Cardiothoracic Surgery, Haukeland University Hospital, Bergen, Norway; Department of Cardiothoracic Surgery, Leiden University Medical Center, Leiden, The Netherlands; Department of Molecular Medicine and Surgery, Karolinska Institutet, Stockholm, Sweden; Department of Cardiothoracic Surgery, Karolinska University Hospital, Stockholm, Sweden; Department of Cardiothoracic Surgery, Leiden University Medical Center, Leiden, The Netherlands; Department of Cardiothoracic Surgery, Rigshospitalet, Copenhagen, Denmark; Department of Cardiothoracic Surgery, Leiden University Medical Center, Leiden, The Netherlands

**Keywords:** Aortic root replacement, Sex difference

## Abstract

**OBJECTIVES:**

To investigate and quantify differences in survival and reinterventions between sexes after aortic root replacement with a stentless bioprosthesis, stratified for preoperative valve lesion.

**METHODS:**

Elective adults undergoing aortic root replacement with the Freestyle bioprosthesis at six North-Atlantic centres were included. Survival analyses were performed using the Kaplan–Meier method or Aalen-Johansen with death as competing risk as relevant. Results were quantified using uni- and multivariable Cox regression tested using a log-rank likelihood ratio test.

**RESULTS:**

In total, 884 patients were analysed for a median follow-up time of 10 years. Females were 4 years older. Survival was significantly worse in females operated for aortic valve insufficiency [60.7% and 72.2% for females and males at 14 years, respectively (*P* = 0.001)], but not for the other indications, even after correction for age. There were no differences in early outcomes or need for reoperation between the sexes and between the different aortic valve pathologies.

**CONCLUSIONS:**

Sex difference in survival outcomes depends on pathology, and females have, compared to males, more symptoms preoperatively regardless of type of valve lesion and worse outcome after aortic root replacement due to aortic insufficiency. Updated surgical risk scores should account for interaction between sex and pathology, and the surgical community must raise awareness on risk of patient’s or doctors delay to surgery.

## Graphical Abstract

**Figure:**
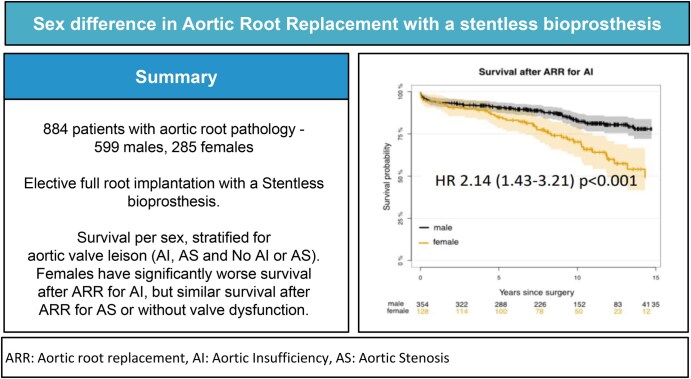

## INTRODUCTION

Cardiovascular disease presents differently in females and males regarding symptoms, pathophysiology, natural history and treatment [1–3]. This is also true for cardiac surgery, where differences in outcomes between males and females have been reported [4, 5], and therefore, female sex is recognized as an independent risk factor for mortality in both the European and American cardiac risk scores [6, 7].

In aortic valve disease patients, differences in pathophysiological processes, including myocardial fibrosis and ventricular hypertrophy, have been observed [8]. On the level of valve pathology, i.e. aortic valve stenosis (AS) or insufficiency (AI), imaging and morphological differences have been described between the sexes [8–11]. This relates to differences in presentation, disease progression and prognosis [12–15]. Consequently, patient sex likely poses different risks for adverse outcomes after cardiac surgery, depending on the pathology leading to surgery.

Studies investigating sex differences after aortic root surgery are scarce. Furthermore, the weight of female sex on the risk associated to the aortic root procedure itself is uncertain. The aim of this study was to use an international multicentre database on the freestyle prosthesis to investigate sex differences in outcomes after aortic root replacement (ARR), quantifying the possible survival difference, and to investigate differences in reintervention, depending on sex and aortic valve pathology.

## PATIENTS AND METHODS

### Ethics statement

The study was conducted in accordance with the Declaration of Helsinki. The study was approved by the Ethics Committee, Copenhagen Denmark (H-16047065), the Swedish Ethical Review Authority (2017/1198–31/2), the Norwegian regional committee for ethical medical and health research (2018/1548/REK vest), The National Bioethics Committee, Iceland (VSN-10–009-V8-S1) and the Medical Ethics Committee of the Leiden University Medical Centre (LUMC) (P14.147), and the need for patient informed consent was waived.

### Study design, inclusion and exclusion

This study was a multicentre, retrospective and observational, merging data from six institutions across Scandinavia and Western-Europe (Supplementary Material, File S1). Medical records were reviewed for baseline characteristics, clinical outcomes and echocardiography reports. Survival status was sourced from national registries and medical records. All adult patients who underwent full root replacement with a Freestyle^®^ stentless bioprosthesis (Medtronic, Minneapolis, MN, USA) in the aortic position between 1995 and 2017, and for Leiden also up to 2021, were included. The surgical technique of root replacement has generally remained stable over the study period and is described in previous publications on the current dataset [16, 17]. Exclusion criteria were patients lost to follow-up within 30 days and operation for dissection or endocarditis. Patients with several Freestyle implantations were included once, only after the first implant. A flowchart of inclusion and exclusion is found in the Supplementary Material (Supplementary Material, Fig. S1).

### Outcomes

The primary outcome was all-cause mortality. Survival status and date of (any) death was found in the medical records which draw information from the population registries. Patients who were alive were noted as followed up until the date of medical record review. Secondary outcomes were aortic valve reintervention and short-term outcomes (reoperation for bleeding or tamponade, cerebrovascular accidents, defined as major or minor stroke transient ischaemic attack, and permanent pacemaker-implantation).

### Stratification for aortic valve dysfunction

To avoid confounding caused by different distribution of valve lesion among males and females, differences in prognosis depending on valve-lesion, and by sex-dependent differences in valve lesion-specific pathology, patients were grouped as (1) aortic valve insufficiency or AI, defined as moderate or severe insufficiency, also in the presence of stenosis, (2) aortic valve stenosis or AS, defined as moderate or severe stenosis of the aortic valve with mild or no accompanying insufficiency and (3) competent aortic valve, defined as none or only mild insufficiency or stenosis.

### Statistical analysis

Statistical analysis was performed according to current recommendations [18–20]. Categorical variables were expressed as *n* (%) and continuous variables as median and interquartile range (IQR). Median follow-up time was calculated both as crude and according to the reverse Kaplan–Meier method [21]. Time-to-event analyses were performed using Kaplan–Meier estimation for survival and Aalen-Johansen with death as competing risk to reintervention, and truncated at the time where all groups had >10% remaining at risk. The event difference was tested using the likelihood ratio- or Greys test, respectively, and quantified with crude and multivariable analysis with the Cox proportional hazard assumption regression. The variables for Cox were chosen with regard to clinical relevance [age, atrial fibrillation, preoperative New York Heart Association (NYHA)-class and reoperative surgery]. Variables with missing <30% were imputated with fully conditional specification and chained equations (Stef van Buuren, 2020; mice: multivariate imputation by chained equations. R package version 3.9.0) with the use of Rubin’s rule, and conditioned on sex, age, atrial fibrillation, preoperative NYHA-class and preoperative surgery. Variables with missing >30% (e.g. renal function and left ventricular function) were deemed beyond use. The proportional hazard assumption was not tested, but the hazard ratio (HR) was viewed as a mean over the study period. *Post hoc*, a sensitivity analysis was performed to examine the consequence calendar time on the results; the dataset was split according to date of surgery before or after the median date (22 November 2012). The resulting six subgroups were plotted with Kaplan–Meier according to valve lesions to compare the study periods' early and late outcome.

## RESULTS

### Patients

In total, 884 elective full ARR patients were included in this study; 599 (68%) were male, and 285 (32%) were female. Eight patients were lost to follow-up within 30 days, and 16 and 38 patients, respectively, were excluded due to an elective operation for chronic dissection or ‘cold’ endocarditis. Forty-nine patients underwent two (*n* = 45) or three (*n* = 4) Freestyle implantations. Males more commonly received an ARR due to AI (55% of male Freestyle-ARR), whereas females were equally likely to undergo surgery due to AI (45% of all female Freestyle-ARR) or AS (46% of all female Freestyle-ARR). A minority of both males and females had competent aortic valves (12% and 9% respectively) (Table 1). Overall, females were significantly older (64 years vs 68 years, *P* < 0.0001) and had slightly worse renal function. We observed equal proportions of previous cardiac surgery, hypertension, diabetes and atrial fibrillation in all groups. Indications for ARR are shown in Table 1.

**Table 1:** Preoperative characteristics

	AI		AS		No AI/AS		All	
Variable Median (Q1, Q3); n (%)	Males *N* = 354	Females *N* = 128	Males *N* = 171	Females *N* = 131	Males *N* = 74	Females *N* = 26	Males *N* = 599	Females *N* = 285
Age [years]	64 (57, 70)	68 (59, 74)	64 (58, 69)	69 (61, 76)	65 (59, 70)	68 (60, 75)	64 (57, 70)	68 (59, 75)
Indication^a^								
Dilatation	289 (91)	87 (76)	95 (62)	46 (39)	42 (81)	10 (67)	426 (82)	143 (58)
Bail-out	1 (0.3)	1 (0.9)	0 (0)	1 (0.9)	0 (0)	0 (0)	1 (0.2)	2 (0.8)
Small root	8 (2.5)	14 (12)	21 (14)	38 (32)	0 (0)	1 (6.7)	29 (5.6)	53 (21)
Other	18 (5.7)	13 (11)	37 (24)	32 (27)	10 (19)	4 (27)	65 (12)	49 (20)
BMI [kg/m^2^]	26.3 (24, 28)	24.8 (22, 29)	27.0 (24, 30)	26.4 (23, 30)	26.9 (24, 29)	25.8 (24, 28)	26.6 (24, 29)	25.7 (23, 29)
NYHA								
I	125 (37)	29 (24)	56 (34)	24 (19)	46 (65)	10 (40)	227 (39)	63 (23)
II	147 (43)	42 (34)	69 (42)	55 (44)	16 (23)	12 (48)	232 (40)	109 (40)
III-IV	68 (20)	52 (42)	39 (24)	46 (36)	9 (12)	3 (12)	116 (21)	101 (37)
Hypertension	219 (62)	83 (65)	96 (56)	75 (59)	42 (57)	14 (54)	357 (60)	172 (61)
Previous cardiac surgery^b^	58 (16)	25 (20)	26 (15)	24 (19)	26 (35)	12 (46)	110 (18)	61 (22)
Previous AVR	44 (12)	21 (16)	18 (11)	20 (16)	21 (28)	10 (38)	83 (14)	51 (18)
Previous CVA	36 (10)	13 (10)	13 (7.6)	10 (7.8)	7 (9.5)	3 (12)	56 (9.4)	26 (9.3)
Atrial fibrillation	55 (16)	21 (16)	18 (11)	12 (9.4)	14 (19)	2 (7.7)	87 (15)	35 (12)
Diabetes	17 (4.8)	9 (7.0)	28 (16)	18 (14)	9 (12)	0 (0)	54 (9.0)	27 (9.6)
COPD	24 (7.3)	12 (9.8)	12 (7.7)	10 (8.7)	7 (9.9)	4 (16)	43 (7.7)	26 (9.9)
Peripheral artery disease	9 (2.5)	5 (3.9)	9 (5.3)	8 (6.3)	2 (2.7)	3 (12)	20 (3.3)	16 (5.7)
Previous revascularization								
None	338 (95)	124 (97)	159 (93)	118 (93)	65 (88)	24 (92)	562 (94)	266 (95)
PCI	9 (2.5)	4 (3.1)	9 (5.3)	7 (5.5)	2 (2.7)	1 (3.8)	20 (3.3)	12 (4.3)
CABG	3 (0.8)	0 (0)	1 (0.6)	1 (0.8)	6 (8.1)	0 (0)	10 (1.7)	1 (0.4)
both	4 (1.1)	0 (0)	2 (1.2)	1 (0.8)	1 (1.4)	1 (3.8)	7 (1.2)	2 (0.7)
Se-Creatinine	87 (74, 100)	76 (63, 89)	86 (76, 100)	70 (61, 79)	89 (75, 95)	75 (66, 81)	87 (75, 99)	73 (62, 84)
eGFR								
>85	112 (46)	17 (22)	52 (55)	21 (27)	24 (45)	5 (28)	188 (48)	43 (25)
51–85	125 (52)	58 (75)	43 (45)	55 (71)	28 (53)	13 (72)	196 (50)	126 (73)
<50	5 (2.1)	2 (2.6)	0 (0)	0 (0)	1 (1.9)	0 (0)	6 (1.5)	2 (1.2)
Dialysis	0 (0)	0 (0)	0 (0)	1 (1.3)	0 (0)	0 (0)	0 (0)	1 (0.6)
Mean pressure gradient [mmHg]	19 (8, 40)	33 (12, 44)	44 (32, 53)	42 (32, 53)	12.9 (8.2, 15.8)	11.3 (8.7, 17.4)	30 (13, 46)	39 (23, 51)
Maximum pressure gradient [mmHg]	34 (16, 72)	61 (24, 82)	69 (53, 83)	67 (55, 83)	22 (18, 31)	23 (16, 29)	53 (26, 75)	63 (44, 81)
Left ventricular function								
Good	129 (56)	58 (68)	64 (62)	57 (75)	33 (62)	16 (76)	226 (58)	131 (72)
Moderate	93 (40)	23 (27)	40 (38)	17 (22)	16 (30)	5 (24)	149 (38)	45 (25)
Poor	5 (2.2)	4 (4.7)	0 (0)	2 (2.6)	4 (7.5)	0 (0)	9 (2.3)	6 (3.3)
Very poor	4 (1.7)	0 (0)	0 (0)	0 (0)	0 (0)	0 (0)	4 (1.0)	0 (0)

a Indication: indications for aortic root replacement; Dilatation: aneurysm involving at least the aortic root; Bail-out attempted surgery was not possible to complete (e.g. valve-sparing root replacement or aortic valve replacement); Small root: aortic valve dysfunction in a root/LVOT with risk of PPM in case of simple aortic valve replacement; Other: remaining indications.

b Previous cardiac surgery: any previous surgery with opening of the pericardium.

AI: aortic insufficiency; AS: aortic stenosis; AVR: aortic valve replacement; BMI: body mass index; CABG: coronary artery bypass grafting; COPD: chronic obstructive pulmonary disease; CVA: cerebrovascular accident; eGFR: estimated glomerular filtration rate; Median (Q1, Q3); *n* (%); No AI/AS: neither aortic insufficiency nor aortic stenosis; NYHA: New York Heart Association functional class; PCI: percutaneous coronary revascularization; Se-Creatinine: serum creatinine.

**Figure 1:**
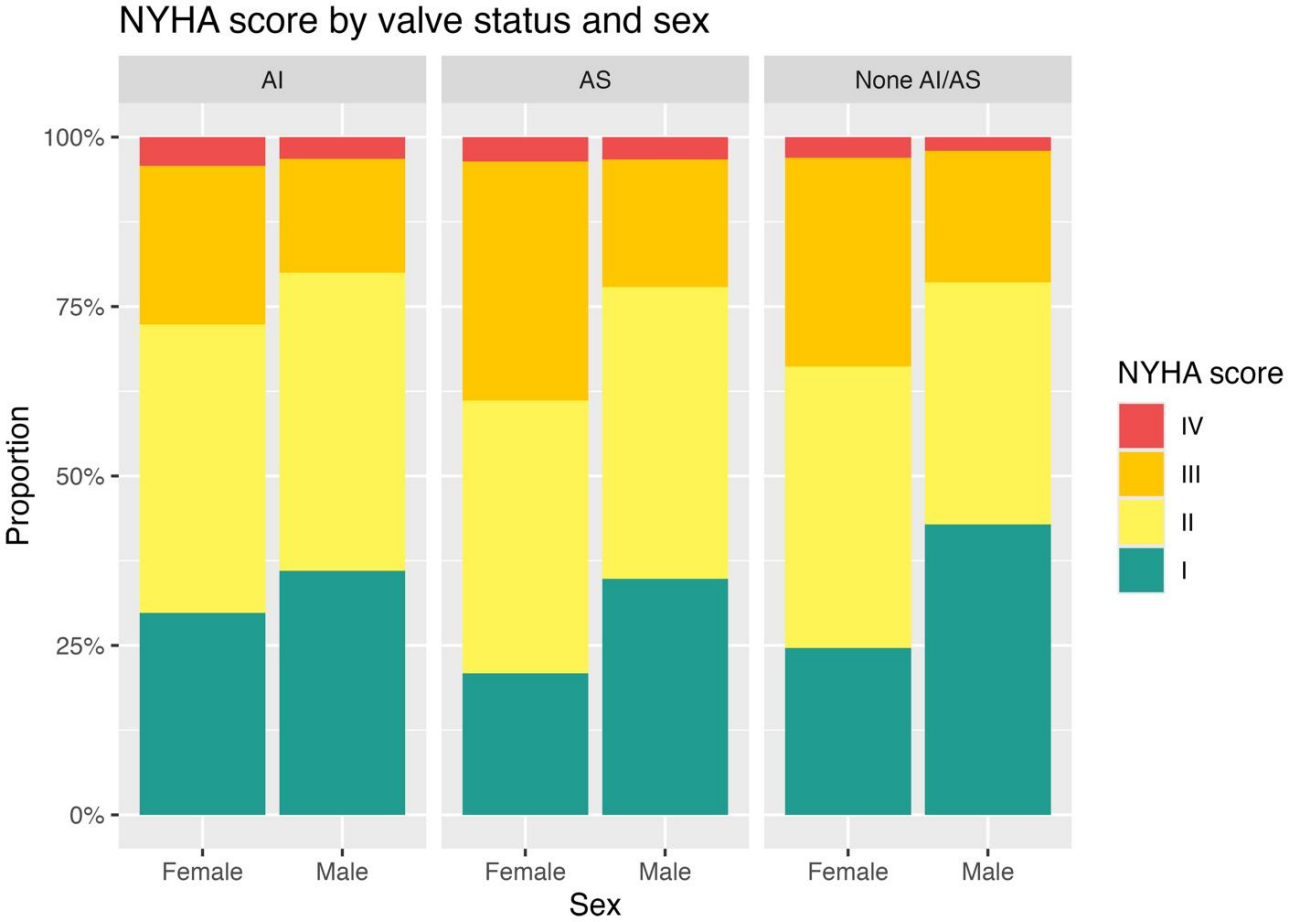
NYHA score stratified by valve status and sex of patients undergoing elective root replacement. AI: aortic insufficiency; ARR: aortic root replacement; AS: aortic stenosis; NYHA: New York Heart Association.

Patients who presented with AI or AS did not differ in preoperative symptoms, but in both categories, females showed more severe symptoms (*P* < 0.001, Fig. 1). For AI, NYHA III/IV was observed twice as often in women compared to males (20.4% vs 41.9%; Fig. 1). Other patient comorbidities did not differ substantially. Patients without valve dysfunction who underwent full root replacement showed significantly better preoperative NYHA scores for both sexes.

### Operative results

The distribution of prosthetic sizes was as can be expected in the patient cohort. There were no clinically relevant differences in perioperative data that are believed to have impacted outcomes. A detailed overview is presented in Table 2.

**Table 2:** Perioperative characteristics

	AI		AS		No AI/AS		All	
Characteristic Median (Q1, Q3); *n* (%)	Males *N* = 354	Females *N* = 128	Males *N* = 171	Females *N* = 131	Males *N* = 74	Females *N* = 26	Males *N* = 599	Females *N* = 285
Freestyle size [mm]								
21	3 (1.3)	13 (16)	1 (0.8)	29 (28)	0 (0)	2 (11)	4 (1.0)	44 (22)
23	8 (3.5)	17 (22)	8 (6.1)	51 (49)	3 (6.0)	4 (21)	19 (4.6)	72 (36)
25	34 (15)	26 (33)	46 (35)	17 (16)	9 (18)	8 (42)	89 (22)	51 (25)
27	78 (34)	19 (24)	45 (34)	5 (4.8)	19 (38)	3 (16)	142 (35)	27 (13)
29	105 (46)	4 (5.1)	32 (24)	2 (1.9)	19 (38)	2 (11)	156 (38)	8 (4.0)
Deep hypothermic circulatory arrest [min]	185 (53)	78 (61)	82 (48)	58 (45)	44 (60)	14 (54)	311 (52)	150 (53)
Antegrade cerebral perfusion [min]	169 (48)	72 (56)	77 (45)	59 (45)	38 (51)	12 (46)	284 (47)	143 (50)
CPB time [min]	174 (140, 208)	154 (125, 220)	184 (147, 227)	174 (136, 213)	188 (153, 257)	183 (150, 220)	177 (144, 216)	167 (132, 215)
Cross clamp time [min]	131 (111, 163)	118 (96, 158)	140 (111, 178)	128 (99, 157)	132 (111, 194)	142 (113, 164)	133 (111, 169)	124 (101, 158)
Circulatory arrest time [min]	21 (19, 26)	29 (23, 35)	23 (22, 31)	32 (19, 44)	28.0 (25, 29)	26.5 (16, 37)	24 (21, 29)	29 (19, 37)
Concomitant surgery								
Coronary bypass grafting	146 (41)	45 (35)	80 (47)	63 (48)	31 (42)	12 (46)	257 (43)	120 (42)
Mitral valve	26 (7.4)	11 (8.7)	8 (4.7)	8 (6.1)	9 (12)	3 (12)	43 (7.2)	22 (7.7)
Tricuspid valve	10 (5.7)	5 (9.6)	4 (4.0)	3 (4.1)	3 (8.1)	0 (0)	17 (5.5)	8 (5.8)
Aortic arch	15 (8.4)	4 (5.3)	3 (4.2)	0 (0)	2 (5.6)	0 (0)	20 (7.0)	4 (2.7)
Ascending aorta	171 (54)	56 (48)	75 (50)	33 (29)	35 (53)	15 (71)	281 (53)	104 (42)

CPB: cardiopulmonary bypass; mm: millimetres; min: minutes.

### Early outcomes

There were no significant differences between groups in early outcomes, including early mortality (Table 3).

**Table 3:** Early postoperative outcomes

	AI			AS			No AI/AS			All		
Characteristic *n* (%); % (95% CI)	Males *N* = 354	Females *N* = 128	*P*	Males *N* = 171	Females *N* = 131	*P*	Males *N* = 74	Females *N* = 26	*P*	Males *N* = 599	Females *N* = 285	*P*
72-hour mortality	1.4 (0.2–2.6)	0.8 (0–2.3)	>0.9	2.3 (0.1–4.6)	3.1 (0.1–6.0)	0.7	0	0	N/A	1.5 (0.5–2.5)	1.8 (0.2–3.3)	0.8
30-day mortality	2.8 (1.1–4.6)	3.9 (0.5–7.3)	0.6	3.5 (0.8–6.3)	6.9 (2.5–11.2)	0.2	2.7 (0–6.4)	0 (0)	>0.9	3.0 (1.6–4.4)	4.9 (2.4–7.4)	0.2
90-day mortality	3.7 (1.7–5.6)	4.7 (1.0–8.3)	0.6	4.7 (1.5–7.8)	7.6 (3.1–12.1)	0.3	4.1 (0–8.5)	3.8 (0–11.2)	1	4.0 (2.4–5.6)	6.0 (3.2–8.7)	0.2
Reoperation for bleeding or tamponade	30 (8.5)	7 (5.5)	0.3	13 (7.7)	11 (8.4)	0.8	7 (9.5)	3 (12)	0.7	50 (8.4)	21 (7.4)	0.6
Cerebrovascular accident	1 (0.3)	1 (0.8)	0.5	2 (1.2)	2 (1.6)	>0.9	0 (0)	1 (4.2)	0.3	3 (0.5)	4 (1.5)	0.2
Renal dysfunction												
Permanent pacemaker implantation	8 (4.6)	4 (5.6)	0.8	7 (10)	2 (3.6)	0.3	1 (2.9)	0 (0)	>0.9	16 (5.8)	6 (4.3)	0.5

### Survival

**Figure 2:**
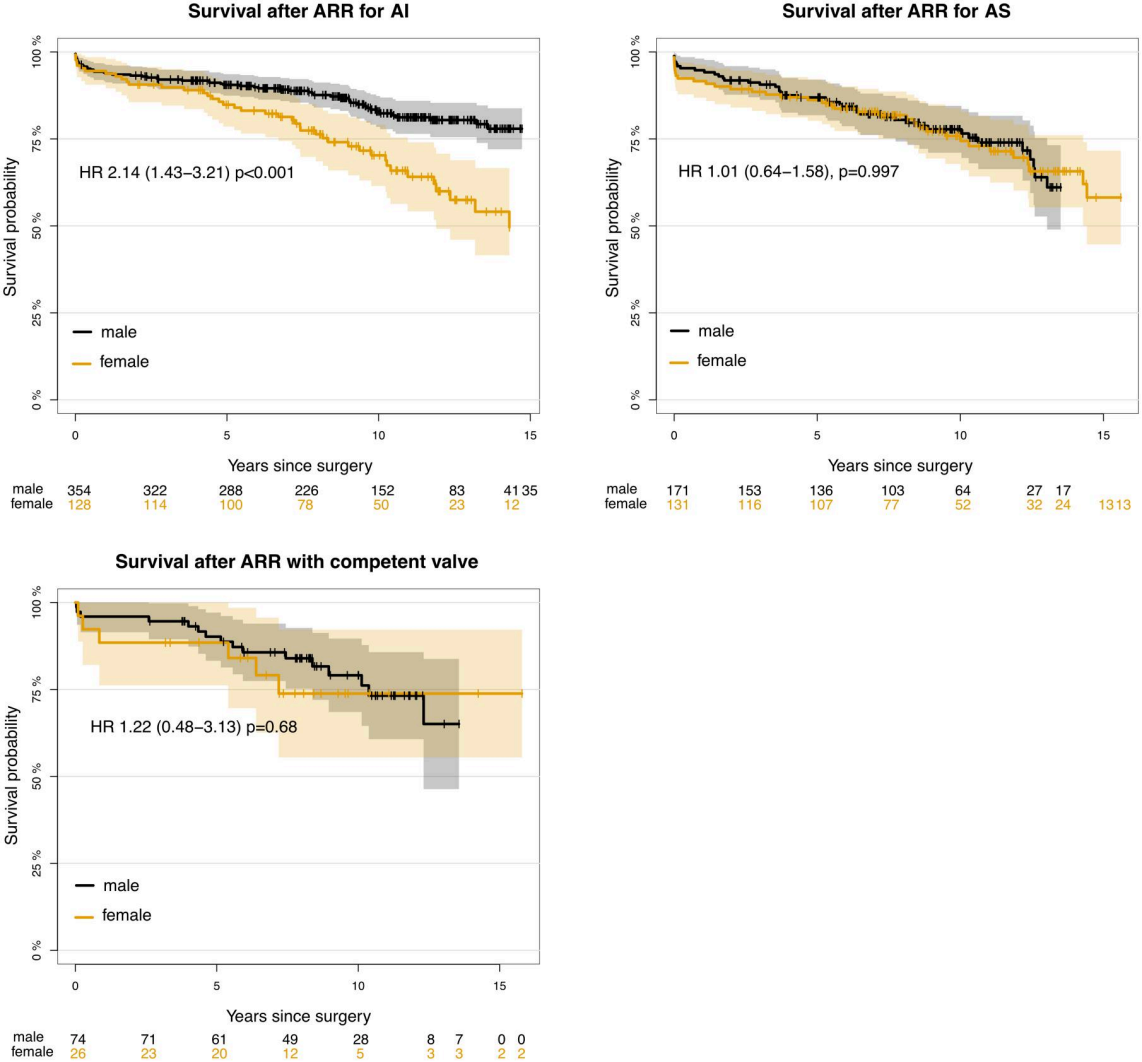
Survival after aortic root replacement with the stentless Freestyle bioprosthesis, per aortic valve lesion. ARR: aortic root replacement; AI: Aortic Insufficiency; AS: aortic stenosis; HR: hazard ratio.

Median follow-up time was 10.1 years for all groups (10.4 and 10.0 years for females and males, respectively, and identical when analysed with the reverse Kaplan–Meier method). Ten percent of population remained at risk at 13.5, 14.7 and 15.8 years for male subgroups and correspondingly at 15.6, 14.3 and 13.6 years for the female subgroups. Over the study period, survival was significantly different in the total cohort (*P* = 0.003). The 14-year survival was 60.7% and 72.2% for females and males, respectively. The difference was driven by the AI-group, with 14-year survival at 54.0% and 77.9% for females and males, respectively (HR 2.14, 95%CI1.43–3.21 *P* < 0.001, Fig. 1). Adjustment for age, atrial fibrillation, preoperative NYHA-class and reoperative surgery reduced the effect to HR 1.71 (1.12–2.62), *P* = 0.01, but it remained significant. The difference in renal function was not included in the analysis because of 36% missing, and furthermore, the difference was not deemed of clinical relevance (very few patients had eGFR < 50). Fourteen-year survival rates in the AS group were 61.1% and 65.7% for females and males, respectively (HR 1.01, 95% CI 0.64–1.58, *P* = 1.00). When adjusted for age, atrial fibrillation, preoperative NYHA-class and reoperative surgery, there was a non-significant trend favouring female survival (HR 0.65, 95% CI 0.40–1.05, *P* = 0.08). In the group with a competent aortic valve, we observed no difference in survival with or without adjusted Cox (Fig. 2).

### Reinterventions

**Figure 3:**
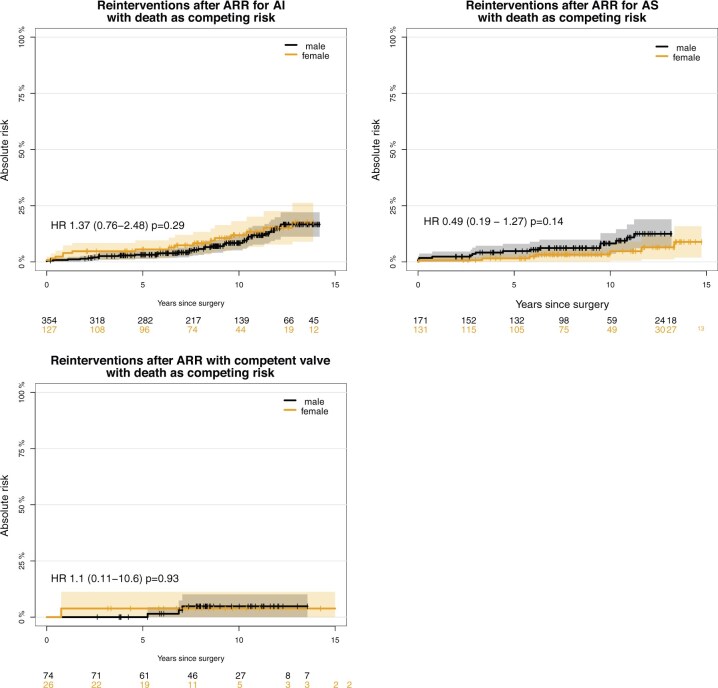
Reinterventions after aortic root replacement with the stentless Freestyle bioprosthesis, per aortic valve lesion. ARR: aortic root replacement; AI: aortic insufficiency; AS: aortic stenosis; HR: hazard ratio.

The unadjusted freedom from reintervention rates at 14 years were 52.2% and 60.0% for females and males, respectively (*P* = 0.2). The occurrence of reinterventions was similar for males and females for all valve lesions. Freedom from reintervention curves with death as a competing risk and related HRs are shown in Fig. 3.

## DISCUSSION

This study describes sex differences in outcomes after elective ARR with a stentless bioprosthesis in real-world data from six European centres. We found that female survival was worse compared to male survival after ARR for aortic valve regurgitation (AR). This difference remained significant when adjusted for age, atrial fibrillation, preoperative NYHA class and redo surgery. Sex-related outcomes were not different in the subgroups with AS or root aneurysm with a competent valve. Reintervention rates were similar for the sexes at 14-years follow-up.

### Survival—AR

Females had worse long-term survival compared to males when operated on for AR. This has previously been described in patients with bicuspid aortic valves and AR but also in females undergoing aortic valve surgery in general [15, 22]. The difference was not explained by symptoms or comorbidities. The lack of difference in both other indications suggests that underlying pathophysiological mechanisms, rather than the procedure itself, is responsible for this survival difference. Interestingly, our group previously found that patient characteristics rather than the procedure or indication determines outcome [16, 17]. Current European and American guidelines on valvular heart disease are clear on the indication for surgery in AI patients when patients are symptomatic [23, 24]. In asymptomatic patients, other factors are to be considered, such as a decrease in left ventricular function or (progressive) left ventricular dilatation. For left ventricular dilatation, both absolute [left ventricular end systolic diameter (LVESD) >50 mm] and indexed (indexed LVESD >20–25 mm/m^2^) cut-off values are maintained in the guidelines. It is known, however, that AI severity and left ventricular dilatation are closely associated in males, but not in females [9]. The question has been raised whether indexing of left ventricular dimensions alone is sufficient in AI-grading in women, or whether different cut-off values or even other parameters should be used [14]. In extension, the question is whether we are able to offer these patients surgery before their prognosis is worsened. In the USA, it has been seen that the previously noticed difference is reduced in modern time, perhaps indicating more awareness on female valve disease [15]. A *post hoc* sensitivity analysis of our results, performed to examine the consequence of the long study period stretching over 20 years, supports this notion (see Supplementary Material, Fig. S2B). This is paralleled in our study where outcomes for females operated for AI in the late study period are indeed improved compared to the early period, and much overlap that of males operated in the early study period. Interestingly, the increased mortality in the early study period manifests at roughly 4 years postoperatively where there is a marked drop in survival for the females operated in the early period. At approximately 8 years, the difference is clearly statistically significant, despite the power loss in the analysis caused by further splitting the subgroups, and it continues to fall drastically. The timing implies relation to (lack of) cardiac recovery, which could well relate to the timing of surgery and consequently irreversible remodelling of the left ventricle, as suggested.

### Survival—AS

There were no differences across groups (Table 3). Females trended towards higher 30-day mortality compared to males (6.9% vs 3.5%, *P* = 0.2), which is in line with several previous reports of female sex as a risk factor for early mortality [12, 25]. However, both 72-hours- and 90 days point-mortality rates are slightly more similar (Table 3), supporting the that no real differences exist. Intermediate survival after ARR for AS in our population is equally good for females as for males. In fact, the adjusted cox regression analysis implied that female sex might potentially be protective, although this was not statistically significant. Interestingly, AS patients in this ARR group received larger valves than isolated aortic valve replacement patients would have. Women with smaller roots and left ventricular outflow tracts may thus benefit from ARR [26]. The underlying pathology may also be different from the ‘classic’ aortic stenosis patients, perhaps in part explaining that we cannot reproduce previous findings of worse outcomes in females [27]. In the sensitivity analysis (Supplementary Material, Fig. S2A), females operated for AS are those that have most improved their outcome from the early to the late study. That could be related to technical circumstances such as a larger effective orifice area in a time where prosthesis-patient mismatch has received more focus. As the Freestyle generally have large openings also in smaller sizes, it may as well be related to timely surgery. Whatever the mechanism, it seems to benefit females even more than males. More diffuse myocardial fibrosis has been observed in females compared to males in patients with AS and may suggest the latter as more likely [8].

### Survival—root aneurysms with well-functioning valves

For the patients operated for root aneurysm without presence of valve dysfunction, there is no evidence of any difference between the sexes. The similar survival corroborates the reports from several studies on aortic surgery and sex [13, 14, 25, 28]. Some report higher in-hospital mortality for women, but only van Kampen reports poorer female survival mid-term [12–14, 25]. Comparison should however be with caution, as the study includes approximately 8% urgent surgery. This may seem little but can contribute with quite a few events both early and mid-term. In the sensitivity analysis, the groups without valve dysfunction are too small to allow much inference. There is no sign of contradictory findings. In all, our and other’s findings support the idea that left ventricular status may be the most important predictor of sex-dependent outcome difference.

### Reinterventions

Sex did not affect the need for reintervention in this study. The adjusted freedom from reintervention rates at 14 years show an expected durability of the prosthesis considering the age of the current cohort. There is no signal that sex or underlying pathology has an impact on valve durability.

### Perspectives

This study aligns with the literature, in that effects of sex on prognosis varies with improved perioperative care, and with preoperative condition. Based upon current data, it is not possible to include sex in risk scores as an independent covariable. It does still seem to play a substantial role in the prediction of outcome. It would be interesting to investigate the interaction between indication and left ventricular functional status. The joint conclusion of the literature and the current study is also that, in contrast to coronary artery bypass grafting, there is very little implication that female sex tolerates aortic root surgery poorer than males. Consequently, it should be possible to remove the sex-dependent difference—most likely by well-defined indication cut-offs that need evaluation and validation in further studies. Although the results are generally improving, the more pronounced symptomatology in females preoperatively implies that patient or doctor’s delay may still contribute to unnecessarily poor outcomes after valve surgery in females.

### Strengths and limitations

This study is strengthened by the large study population and the multicentre design, thus delivering real-world data. Its retrospective design however comes with the disadvantages of limited availability of data. Furthermore, the study period ranges over more than 20 years of surgery, with changes in trends for diagnosis, referral, acceptance for surgery and perioperative care. To assess the consequence of this time period on the results, a sensitivity analysis is presented in the Supplementary Material and taken into consideration in the discussion and conclusion of the study.

## CONCLUSION

In conclusion, females who underwent ARR with a stentless bioprosthesis for AI displayed worse long-term prognosis compared to male patients. This effect was driven by female patients operated earlier in the study period and reduced in later operated patients. There is no difference between the survival of the sexes when operated for aortic stenosis, or for root dilatation with a well-functioning valve. Surgical timing and risk varies between sexes and preoperative conditions which should to be reflected in updated guidelines and risk scores.

## Supplementary Material

ezaf161_Supplementary_Data

## Data Availability

Due to local legislations, data access is strictly limited to the authors specifically as regulated by authority approvals, and therefore not possible to share.

## ABBREVIATIONS

AI: Aortic valve insufficiency
AR: Aortic valve regurgitation
ARR: Aortic root replacement
AS: Aortic valve stenosis
HR: Hazard ratio
NYHA: New York Heart Association
