# Supplementary figures and images for: Sex difference in aortic root replacement with a stentless bioprosthesis
Source: Eur J Cardiothorac Surg. 2025 May 12;67(6):ezaf161. doi: 10.1093/ejcts/ezaf161 (PMC12148212; doi:10.1093/ejcts/ezaf161)

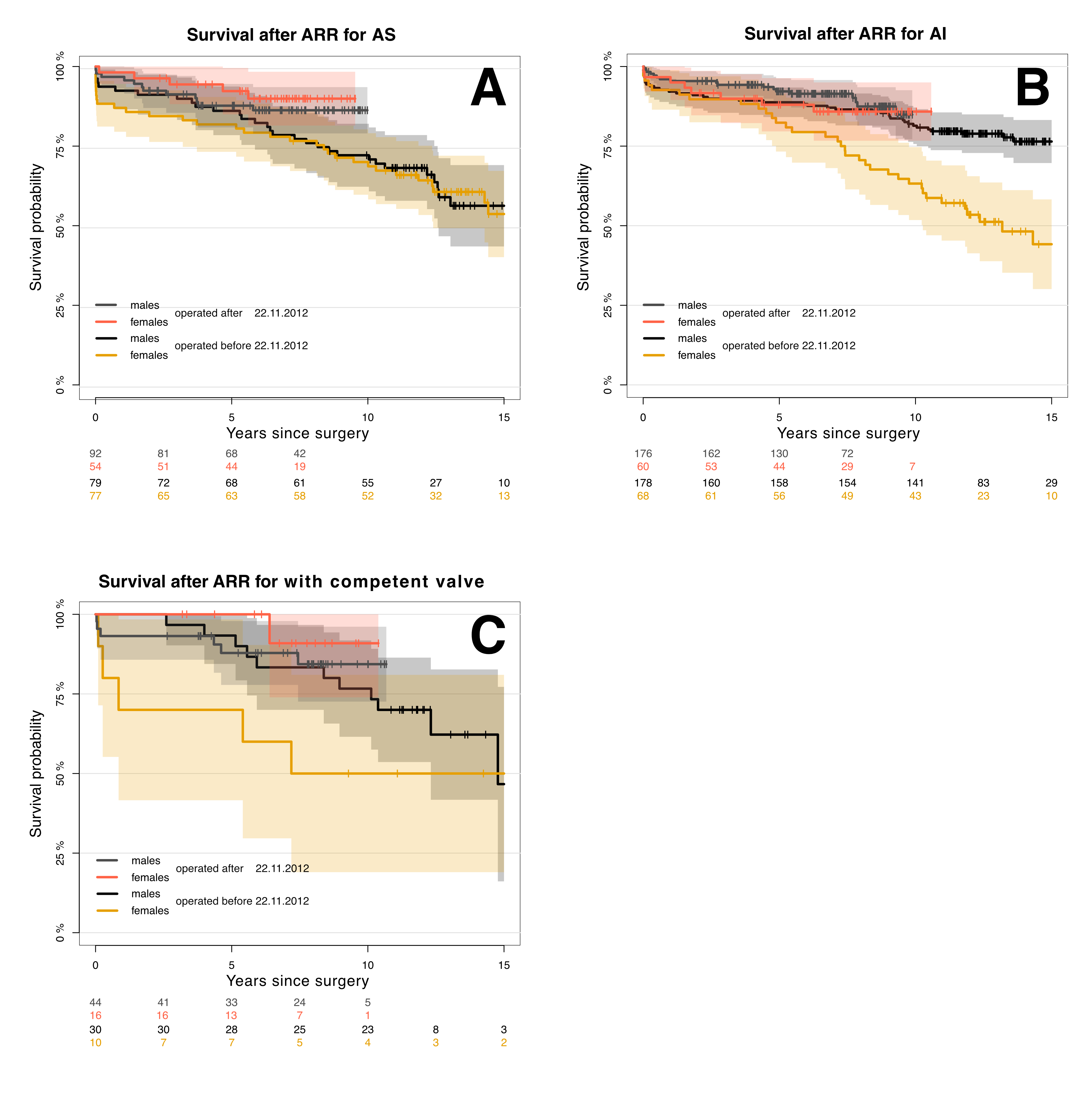

Supplement: ezaf161_Supplementary_Data [file ezaf161_supplementary_data.zip › fnac_sex_survbycalendartime.png]
