# Supplementary material for: Sex difference in aortic root replacement with a stentless bioprosthesis
Source: Eur J Cardiothorac Surg. 2025 May 12;67(6):ezaf161. doi: 10.1093/ejcts/ezaf161 (PMC12148212; doi:10.1093/ejcts/ezaf161)
Supplement: ezaf161_Supplementary_Data [file ezaf161_supplementary_data.zip › Supplemental_figure.zip!/Freestyle North Atlantic Collaborative Contributors 241107.docx]

Freestyle North Atlantic Collaborative Contributors

- per center

| *Leiden* |  |
| --- | --- |
| Jesper Hjortnæs | Adriaan Schneider |
| Robert Klautz | Patrick Timmemanns |
| Jerry Braun | Alex Cotovanu |
|  |  |
| *Copenhagen* |  |
| Morten Smerup^2a+b,3^ | Hanna Dagnegård^2a,3,4^ |
| Nikolaj Ihlemann^5^ | Kirstine Bekke^2^ |
| H Gustav H Thyregod^2a+b^ | Jannie Lind^2^ |
| Jens T. Lund^6^ |  |
|  |  |
| *Stockholm* |  |
| Ulrik Sartipy^7,8^ | Nathalie Glaser^7,8,9^ |
|  |  |
| *Bergen* |  |
| Rune Haarverstad^10^ | Solveig Moss Kolseth^10^ |
|  | Jørgen Brekke Vennemo ^10^ |
|  | Asbjørn Lie ^10^ |
|  |  |
| *Linköping* |  |
| Farkas Vanky^11^ | Christoffer Wallén^11^ |
|  |  |
| *Reykavijk* |  |
| Tomas Gudbjartsson^12^ | Hörður Ingi Gunnarsson, MD^12^ |
|  |  |
| *Aalborg* | *(statistical support)* |
| Jan Brink Valentin^13^ |  |
| Søren Paaske Johnsen^13^ |  |

1 Department of Cardiothoraic Surgeyr, Leiden University Medical Center, Leiden, The Netherlands

2 Department of Cardiothoracic Surgery, Rigshospitalet, University Hospital of Copenhagen, Copenhagen, Denmark

a division of complex adult cardiac surgery

b division of congenital cardiac surgery

3 Department of Cardiac-, Lung- and Vascular Surgery, T, Odense University Hospital, Odense, Denmark, Division of cardiac surgery

4 Department of Clinical Medicine, Copenhagen University, Copenhagen, Denmark

5 Department of Cardiology, Bispebjerg Hospital, Copenhagen, Denmark

6 Department of Cardiothoracic Surgery, Auckland University Hospital, New Zealand

7 Department of Molecular Medicine and Surgery, Karolinska Institutet, Stockholm, Sweden

8 Department of Cardiothoracic Surgery, Karolinska University Hospital, Stockholm, Sweden

9 Department of Cardiology, Södersjukhuset, Stockholm, Sweden

10 Department of Heart Disease, Haukeland University Hospital and University of Bergen, Bergen, Norway

11 Department of Cardiothoracic and Vascular Surgery, Heart Centre, Department of Health, Medicine and Caring Sciences, Linköping University, Linköping, Sweden, division of cardiac surgery

12 Department of Cardiothoracic Surgery, Landspítali, University Hospital, Faculty of Medicine, University of Iceland, Reykjavik, Iceland

13 Danish Center for Clinical Health Services Research (DACS), Department of Clinical Medicine, Aalborg University and Aalborg University Hospital, Aalborg, Denmark
