# Supplementary material for: Sex difference in aortic root replacement with a stentless bioprosthesis
Source: Eur J Cardiothorac Surg. 2025 May 12;67(6):ezaf161. doi: 10.1093/ejcts/ezaf161 (PMC12148212; doi:10.1093/ejcts/ezaf161)
Supplement: ezaf161_Supplementary_Data [file ezaf161_supplementary_data.zip › Supplemental_figure.zip!/Supplemental figure 2.pdf]

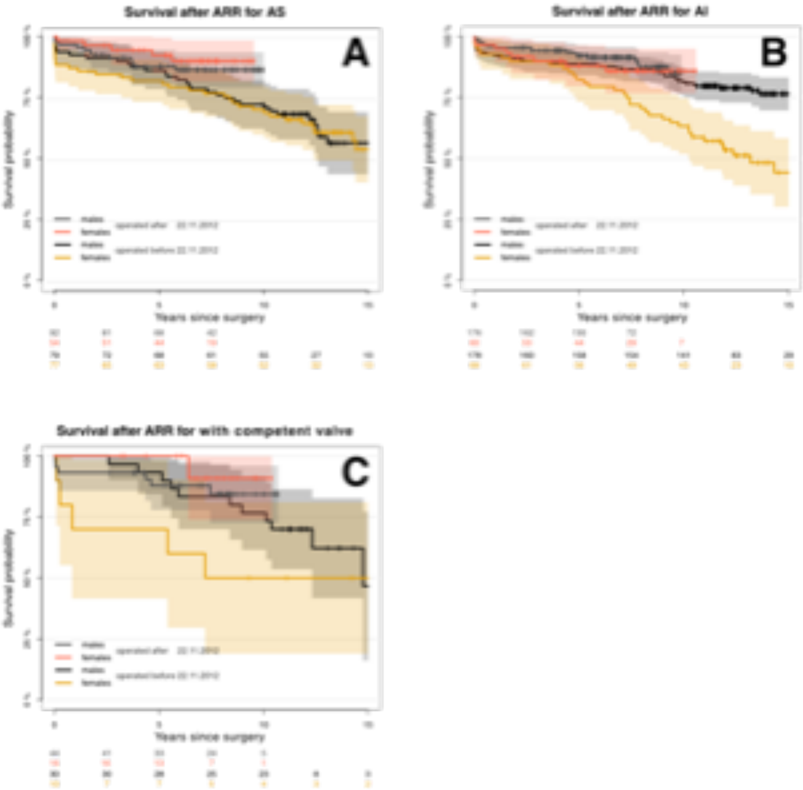

Survival after ARR for AS
A
Survival after ARR for AI
B
Survival after ARR for with competent valve
C
Survival probability
Years since surgery
